# Development and Validation of a Novel Recurrence Risk Stratification for Initial Non-Muscle Invasive Bladder Cancer in the Han Chinese Population

**DOI:** 10.7150/jca.38649

**Published:** 2020-01-14

**Authors:** Zhiyong Wang, Wansheng Gao, Jian Li, Tianen Wang, Man Zhu, Yu Duan

**Affiliations:** 1 Department of Urology, the First Affiliated Hospital of Zhengzhou University, Zhengzhou, Henan 450052, P. R. China.; 2 Department of Clinical Laboratory & Center for Gene Diagnosis, Zhongnan Hospital of Wuhan University, Wuhan, Hubei 430000, P. R. China; 3 Department of Clinical Laboratory, the First Affiliated Hospital of Zhengzhou University, Zhengzhou, Henan 450052, P. R. China.

**Keywords:** Non-muscle invasive bladder cancer, Recurrence, Risk factor, Sensitivity, Specificity

## Abstract

**Background**: Some classification models for determining the risk of recurrence after transurethral resection of bladder tumor (TURBT) in patients with non-muscle invasive bladder cancer (NMIBC) had some shortcomings in clinical applications. This study aimed to investigate whether the European Organization for Research and Treatment of Cancer (EORTC) risk stratification was useful to predict the recurrence of NMIBC in the Han Chinese population. In addition, we developed and validated a novel risk stratification method for recurrence prediction of NMIBC.

**Methods**: Excluding cases who do not meet the inclusion criteria, 606 patients with NMIBC from the First Affiliated Hospital of Zhengzhou University were included in the testing and validation groups. The recurrence-free survival (RFS) curve according to the EORTC risk classifications was calculated by the Kaplan-Meier and the log-rank test methods. Receiver operating characteristic (ROC) curve analysis was used to estimate the diagnosis value for recurrence. We built a logistic regression model for recurrence in NMIBC patients combining the independent recurrence prognostic factors. One external validation group including 166 patients with NMIBC from the Zhongnan Hospital of Wuhan University was also used to assess the logistic regression model.

**Results**: There was no significant difference in RFS rates between the groups grouped according to EORTC. We constructed a novel risk model to predict recurrence by classifying patients into two groups using ten independent prognostic factors [bladder cancer-specific nuclear matrix protein 4 (BLCA-4), bladder tumour antigen (BTA), nuclear matrix protein 22 (NMP22), carcinoembryonic antigen (CEA), body mass index, smoking, family history of bladder cancer, occupational exposure to aromatic amine chemicals, number of tumours, bladder instillation of chemotherapeutic agents] to predict tumour recurrence based on logistic regression analyses (testing group). According to the novel recurrence risk classification, there was a significant difference in 5-year RFS rates between the low-risk group and the high-risk group (Validation group and the external validation group).

**Conclusions**: Our novel classification model can be a useful tool to predict recurrence risk in the Han Chinese population with NMIBC.

## Introduction

Bladder cancer has high morbidity and mortality worldwide, with nearly 549,393 newly diagnosed cases and approximately 199,922 patients dying each year 1. In recent years, with the increase of tobacco consumption, and the development of industrialization, the incidence of bladder cancer has been increased year by year 2-3. In China, the incidence and mortality of bladder cancer heads the list among urinary malignant tumors 4. The incidence of bladder cancer in China increased by 56.59% from 1998 to 2008, and the annual growth rate during the 10-year period was 4.6% 4. According to the 2009 diagnostic criteria of the Union for International Cancer Control (UICC), about 75% of bladder tumors are non-muscle invasive bladder cancer (NMIBC), these bladder tumors are confined to mucosal (T_a_ or T_is_) or sub-mucosal connective tissue (T_1_) 5. The remaining 25% are muscle-invasive bladder cancers (MIBCs). NMIBC mainly adopts transurethral resection of bladder tumor (TURBT), which is the first choice for the treatment. TURBT has the characteristics of less bleeding and quick recovery after the operation. However, although the prognosis of NMIBC is generally favorable (5-year survival rate is higher than 80%), 50-80% of patients have an intravesical recurrence after TURBT 6. Early identification of high-risk groups of NMIBC recurrence helps to give effective treatment interventions, and is of great significance to improve the survival rate of patients.

The risk assessment predictive model consists of different prognostic variables that are primarily derived from the natural history of the disease, physical examination, pathological assessment, or biomarkers. When different variables are combined into the model, they can be used to assess the likelihood of a particular event occurring 7. Accurate prediction of the risk of recurrence in NMIBC patients, then developing the best-individualized treatment plan may help us develop the best monitoring program for newly diagnosed and relapsed patients. The European Organization for Research and Treatment of Cancer (EORTC) 8 and the Spanish Urological Club for Oncological Treatment (CUETO) 9 are two important risk assessment predictive models for predicting the risk of NMIBC recurrence. Among them, EORTC is the most commonly used worldwide for risk stratification. Through the EORTC risk group stratification, urologists can predict the risk of short-term or long-term recurrence after TURBT in NMIBC patients to help clinicians decide on treatment and follow-up. EORTC risk stratification divides NMIBC patients into low-risk, intermediate-risk, and high-risk recurrence groups, and then develops different treatment options through these groups 8. However, there are certain restrictions on the EORTC, including the following aspects 10-12: (1) The calculation steps of the EORTC are cumbersome; (2) No tumor heterogeneity was considered; (3) There is no evidence that EORTC can improve patient survival; (4) During the EORTC building process, TURBT did not receive intravesical instillation chemotherapy in 22% of patients, and only 6% of patients received postoperative intravesical Bacillus Calmette-Guerin (BCG) immunotherapy. Moreover, the effect of BCG instillation or intravesical instillation chemotherapy on recurrence was not considered; (5) Less than one-third of patients received immediate intravesical instillation after surgery, and fluorescent cystoscopy was not available at the time. For these reasons, the recurrence rate reported by the EORTC may be too high compared to current clinical treatment practices, and requiring external data validation to confirm its effectiveness. At the same time, due to the advanced treatment methods, the EORTC risk group stratification needs to be adjusted to improve the accuracy of predicting the recurrence risk.

In this study, we applied the EORTC risk group stratification to predict the recurrence of NMIBC in the Chinese Han cohort. In addition, we developed a novel recurrence risk stratification based on clinicopathological characteristics, urine biomarkers and life-history traits to easily estimate the recurrence risk in NMIBC patients after TURBT and validated this novel classification using two validation cohorts.

## Materials and Methods

### Inclusion and Exclusion Criteria

In the testing and validation groups, we analyzed data from patients with NMIBC who underwent initial TURBT at the Department of Urology, First Affiliated Hospital of Zhengzhou University between 2010 and 2014. In the external validation group, we analyzed data from patients with NMIBC who underwent initial TURBT from the Zhongnan Hospital of Wuhan University between 2012 and 2014. The inclusion criteria for patients are: (1) Intraoperative pathology confirmed as NMIBC; (2) The patient who underwent initial TURBT; (3) The clinical stage is T_a_ or T_1_, and single or multiple NMIBC, and no clinical metastasis; (4) Patients undergoing standard intravesical instillation chemotherapy and regular cystoscopy; (5) Patients who completed follow-up after surgery; (6) Patient who has provided informed consent; (7) The general condition is good, no serious heart, lung, liver, kidney and other complications; (8) The patient was conscious and had no history of mental illness. Patients with any of the following were excluded: (1) Follow-up time is less than three months; (2) History of cancer; (3) History of radiotherapy and chemotherapy treatment; (4) History of BCG therapy; (5) Patients or their families are reluctant to participate in this study; (6) The patient's clinical pathology data is incomplete. To achieve accurate pathological staging, complete resection was gathered including the muscle layer of the bladder wall. The TNM classification of NMIBC was assessed based on the Guidelines of the European Association of Urology 13. The grade was classified according to the 2004 World Health Organization classifications in NMIBC (based on a grading system published in 1998) 14.

### Collection of clinicopathological and life history variables

The clinicopathological data, including age, sex, body mass index, hypertension, diabetes, smoking, drinking, histologic type, differentiation status, pathological grade, depth of invasion, metastatic status size and number of tumours, intravesical therapy, presence of concomitant carcinoma *in situ* (CIS), were all obtained. Each of these variables adhered to the EORTC scoring system 8. Life history variables, including smoking, drinking, family history, occupational exposure to aromatic amine chemicals (aniline, diaminobiphenyl, 2-naphthylamine, 1-naphthylamine), were also collected.

### Urine sample collection and biomarkers detection

Urine samples from NMIBC patients were separately collected before discharge. Urine biomarkers tested in this study include bladder cancer-specific nuclear matrix protein 4 (BLCA-4), bladder tumor antigen (BTA), nuclear matrix protein 22 (NMP22), and carcinoembryonic antigen (CEA). In order to reduce the impact of urine specific gravity on the test results, creatinine-corrected total urinary biomarker concentration was estimated by dividing the total urinary biomarker concentration by the creatinine concentration 15.

### Patients follow-up, recurrence-free survival (RFS) and EORTC risk classification

All recurrences were confirmed by histopathology and removed by TURBT or biopsy. Patients who did not relapse or die were examined on the last date of follow-up. All patients were followed for five years. RFS was defined as the period between the initial TURBT and recurrence. A total recurrence score for each patient was calculated based on the EORTC scoring system 16. Patients were then divided into three risk groups (Low-risk, Intermediate-risk and High-risk) for recurrence 13.

### Testing group, validation group and the external validation group

Testing group: three hundred and sixty patients with initial NMIBC between April 2008 and January 2012 were included. Validation group: two hundred and forty six patients with initial NMIBC between February 2012 and June 2014 were included. The external validation group: one hundred and sixty six patients with initial NMIBC between August 2012 and May 2014 were included. Our study was approved by the Ethics Committee of the First Affiliated Hospital of Zhengzhou University, Zhengzhou, China (2008017) and the Ethics Committee of the Zhongnan Hospital of Wuhan University, Wuhan, China (KS2012-29). Written informed consent was provided in accordance with the ethical principles of the Declaration of Helsinki.

### Logistic regression model establishment and validation

A regression formula for the NMIBC recurrence prediction was established based on the testing group. The formula is: Logit (P) = A_0_+A_1_B_1_+A_2_B_2_+A_3_B_3_+…+A_n_B_n_=ln[p/(1-p)], “p” means the incident probability (NMIBC recurrence), “n” means the number of interference variable, “A” means the influence coefficient of each interference variable, “B” means the value of each interference variable. The validation group was used to assess the above logistic regression model.

### Statistical analysis

All statistical analyses were performed using SPSS version 19.0 (SPSS, Chicago, Illinois). Data were presented as the mean ± standard deviation (SD, normally distributed numeric variables), or median (interquartile range [IQR], non-normally distributed variables), or number of cases (%, counting data). Univariate and multivariate models adjusted for possible recurrence variables (age, sex, BMI, hypertension, diabetes, smoking, drinking, histologic type, differentiation status, pathological grade, depth of invasion, metastatic status size, pathological grade, size and number of tumours, intravesical therapy, presence of CIS, smoking, drinking, family history, occupational exposure to aromatic amine chemicals, BLCA-4, BTA, NMP22, and CEA) were performed to investigate the relation of various variables with the recurrence in testing group. RFS curves were calculated by the Kaplan-Meier and the log-rank test methods. Receiver operating characteristic (ROC) curve analysis was used to estimate the differential diagnosis values for recurrence, and the results were reported as the area under the curve (AUC).* P*-values <0.05 was considered significant.

## Results

### Patient characteristics in the testing group

**Fig 1:**
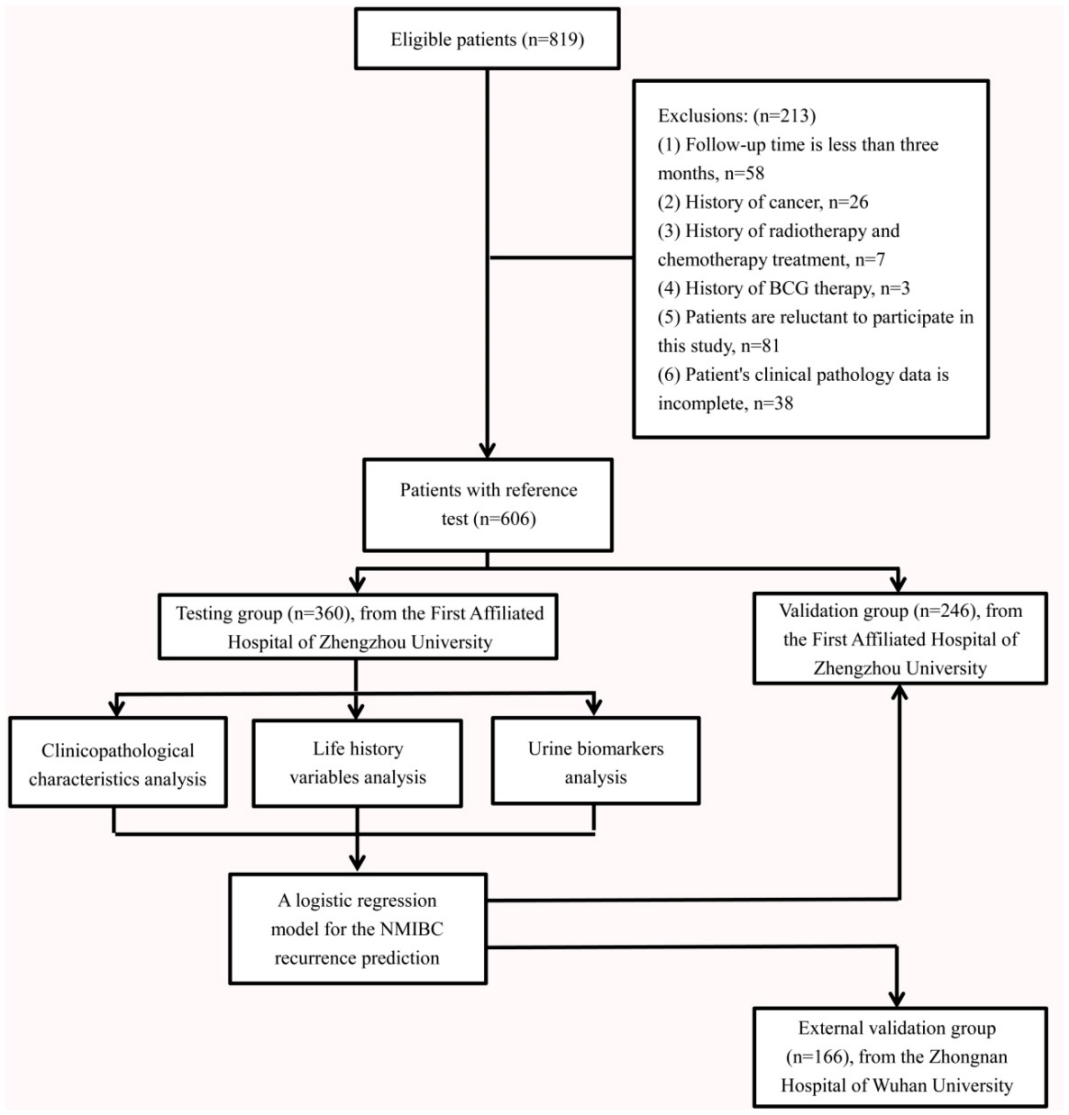
Flow diagram shows the inclusion and exclusion of eligible patients.

From 2008 and 2014, we identified 819 eligible patients at the First Affiliated Hospital of Zhengzhou University. All patients were Chinese. Excluding cases who do not meet the inclusion criteria, 606 were included in the final analysis (Fig. 1). There were 360 patients with initial NMIBC (male; n=288, 80.0%, female; n=72, 20.0%) in the testing group. The patients' creatinine corrected urine biomarkers (BLCA-4, BTA, NMP22 and CEA), clinicopathological characteristics and life-history traits are presented in Table 1. The median follow-up periods were 44.5 months (IQR: 8.5-60.0). The median age was 70 years old (IQR: 62-79). According to the EORTC risk group stratification (Table S1), the intermediate-risk group had predominantly higher number of cases (n=287; 79.7%) compared with the low-risk (n=31; 8.6%) and high-risk groups (n=42; 11.7%).

### RFS rates stratified by the EORTC risk group stratification in testing group

**Fig 2:**
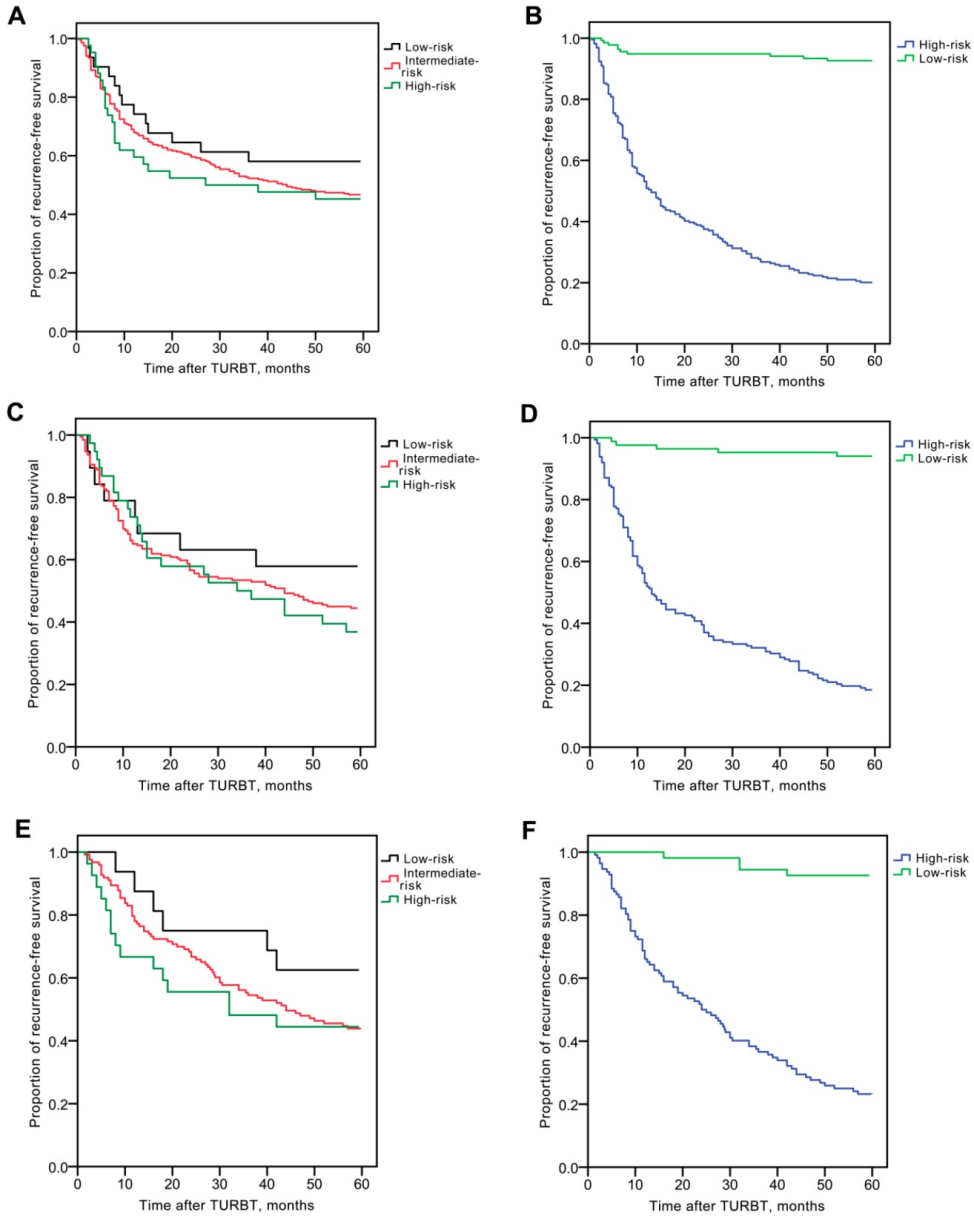
Kaplan-Meier RFS curves, stratified by the EORTC recurrence risk classification or novel recurrence classification in the testing group and validation group. (A) There were no significant differences in RFS rates between groups according to the EORTC risk group stratification in the testing group. (B) There were significant differences in 5-year RFS rates between groups according to the novel recurrence classification in the testing group. (C) There were no significant differences in RFS rates between groups according to the EORTC risk group stratification in the validation group. (D) There were significant differences in 5-year RFS rates between groups according to the novel recurrence classification in the validation group. (E) There were no significant differences in RFS rates between groups according to the EORTC risk group stratification in the external validation group. (F) There were significant differences in 5-year RFS rates between groups according to the novel recurrence classification in the external validation group.

During the follow-up period of the testing group, 189 of the 360 patients (52.5%) experienced intravesical recurrence. Overall, RFS rates were 243/360 (67.5%) at 1 year, 213/360 (59.2%) at 2 years, 189/360 (52.5%) at 3 years, 177/360 (49.2%) at 4 years, and 171/360 (47.5%) at 5 years. The RFS rates at 5 years were 18/31 (58.1%) for the low-risk group, 134/287 (46.7%) for the intermediate-risk group and 19/42 (45.2%) for high-risk group (Fig. 2A). There were no significant differences in RFS rates between groups according to the EORTC risk group stratification (All: *χ^2^*= 1.482, *P* = 0.477; low *vs*. intermediate-risk: *χ^2^*= 1.224, *P* = 0.269; low *vs.* high-risk: *χ^2^*= 1.416, *P* = 0.234; intermediate *vs.* high-risk:* χ^2^*= 0.142, *P* = 0.707).

### The relationship between urine biomarkers, clinicopathological characteristics and life-history traits of NMIBC and their differential diagnosis value for the recurrence of NMIBC

**Fig 3:**
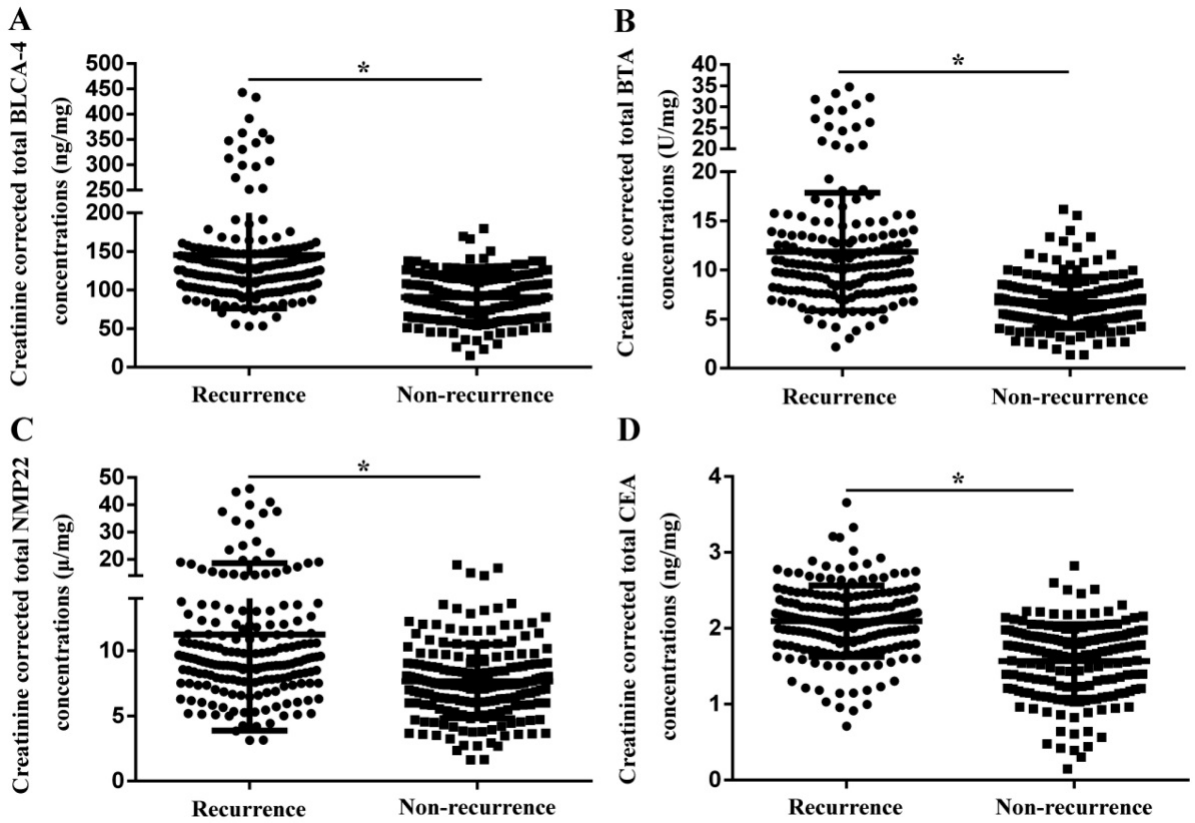
Creatinine-corrected total urinary biomarker concentrations in the recurrence group and the non-recurrence group. (A) BLCA-4. (B) BTA. (C) NMP22. (D) CEA.^ *^ *P*<0.05.

In order to reduce the impact of urine specific gravity on the test results, creatinine-corrected total urinary biomarker concentration was estimated. As shown in Fig. 3, creatinine corrected total BLCA-4 (145.3±69.0 ng/mg *vs.* 90.5±31.3 ng/mg), BTA (11.9±6.0 U/mg *vs.* 6.7±2.6 U/mg), NMP22 (11.2±7.4 μg/mg *vs.* 7.6±2.8 μg/mg) and CEA (2.1±0.5 ng/mg *vs.* 1.6±0.5 ng/mg) concentrations were significantly increased in the recurrence group compared with the non-recurrence group (*P*<0.05).

**Fig 4:**
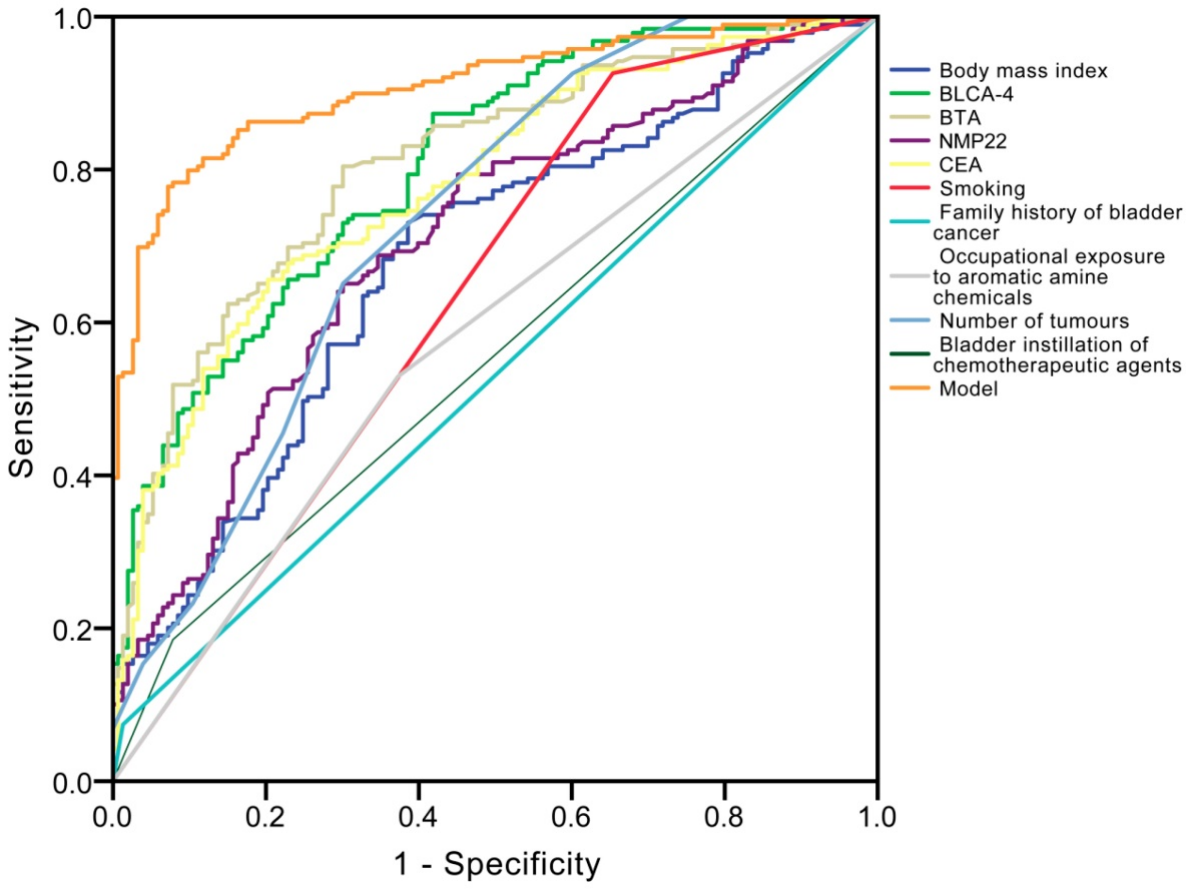
Receiver operating characteristic (ROC) curve analysis for the differential diagnosis values in the ten independent prognostic factors [bladder cancer specific nuclear matrix protein 4 (BLCA-4), bladder tumour antigen (BTA), nuclear matrix protein 22 (NMP22), carcinoembryonic antigen (CEA), body mass index, smoking, family history of bladder cancer, occupational exposure to aromatic amine chemicals, number of tumours, bladder instillation of chemotherapeutic agents] for recurrence.

Univariate and multivariate logistic regression analyses (Table 2) revealed that creatinine corrected total BLCA-4 (*P*<0.001), BTA (*P*<0.001), NMP22 (*P*<0.001), CEA (*P*=0.002) concentrations, body mass index (*P*=0.007), smoking (*P*<0.001), family history of bladder cancer (*P*<0.001), occupational exposure to aromatic amine chemicals (*P*<0.001), number of tumours (*P*=0.010), and bladder instillation of chemotherapeutic agents (*P*<0.001) had significant influence on recurrence. The ROC curve analysis showed that the area under the curve (AUC) of BLCA-4, BTA, NMP22, CEA, body mass index, smoking, family history of bladder cancer, occupational exposure to aromatic amine chemicals, number of tumours, and bladder instillation of chemotherapeutic agents were 0.804, 0.807, 0.705, 0.780, 0.680, 0.636, 0.531, 0.578, 0.731 and 0.553 in the recurrence group, respectively (Fig. 4). The detailed information of diagnostic performances in the testing group is listed in Table 3.

### A novel risk classification predicting recurrence of NMIBC in the testing group

Each of the abovementioned variables with a significant difference in the multivariate model was included in the multivariate logistic regression model. The final risk classification predicting model for NMIBC recurrence prediction was: Logit (P) = 19.379-0.036(BLCA-4)-0.463(BTA)-0.104(NMP22)-1.751(CEA)-0.238(body mass index)-0.872(smoking, yes=1; no=0)-1.174(family history of bladder cancer, yes=1; no=0)+1.660(occupational exposure to aromatic amine chemicals, yes=1; no=0)-0.345(number of tumours)-0.510(bladder instillation of chemotherapeutic agents, yes=1; no=0). The identification value of this model was high with AUC of 0.907 (Fig. 4 and Table 3), and the probability was 0.508, which means if the probability was <0.508, it was classified as high-risk recurrence group, on the contrary, it was classified as low-risk recurrence group.

In this novel recurrence risk classification, 224 cases (62.2%) were in the high-risk group and 136 (37.8%) were in the low-risk group. The RFS rates at 5 years were 45/224 (20.1%) for the high-risk group and 126/136 (92.6%) for the low-risk group. There were significant differences in 5-year RFS rates between the two groups (*χ^2^*= 166.975, *P* < 0.001, Fig. 2B).

### The validation of our novel risk classification predicting recurrence for NMIBC

We included 246 patients with initial NMIBC (male; n=194, 78.9%, female; n=52, 21.1%) in the validation group. The patients' traits are presented in Table 1. Creatinine corrected total BLCA-4, BTA, NMP22 and CEA concentrations were shown in Figure S1. Overall, RFS rates in the validation group were 166/246 (67.5%) at 1 year, 141/246 (57.3%) at 2 years, 132/246 (53.7%) at 3 years, 116/246 (47.2%) at 4 years, and 109/246 (44.3%) at 5 years. According to the EORTC risk group stratification, the intermediate-risk group still had predominantly higher number of cases (n=189; 76.8%) compared with the low-risk (n=19; 7.7%) and high-risk groups (n=38; 15.5%). However, there were no significant differences in RFS rates between groups according to the EORTC risk group stratification (All: *χ^2^*= 1.256, *P* = 0.534; low *vs*. intermediate-risk: *χ^2^*= 0.897, *P* = 0.344; low *vs.* high-risk: *χ^2^*= 1.309, *P* = 0.253; intermediate *vs.* high-risk:* χ^2^*= 0.220, *P* = 0.639, Fig 2C). According to this novel recurrence-risk classification, 84 cases (34.1%) and 162 cases (65.9%) in the validation group were classified into low and high-risk groups, respectively. The RFS rates at 5 years were 132/162 (81.5%) for the high-risk group and 5/84 (6.0%) for the low-risk group. There were significant differences in the 5-year RFS rates between the low-risk group and high-risk groups (*χ^2^*= 114.861, *P* < 0.001, Fig 2D).

The validity of our logistic regression model was also assessed in an external validation group from the Zhongnan Hospital of Wuhan University. The patients' creatinine corrected urine biomarkers (BLCA-4, BTA, NMP22 and CEA), clinicopathological characteristics and life-history traits are presented in Table 1. There were 166 subjects in the external validation group. The median follow-up periods were 41.0 months (IQR: 8.0-60.0). The median age was 74 years old (IQR: 64-82). According to the EORTC risk group stratification, the intermediate-risk group had predominantly higher number of cases (n=123; 74.1%) compared with the low-risk (n=16; 9.6%) and high-risk groups (n=27; 16.3%). However, there were no significant differences in RFS rates according to the EORTC risk group stratification (All: *χ^2^*= 2.125, *P* = 0.346; low *vs*. intermediate-risk: *χ^2^*= 1.880, *P* = 0.170; low *vs.* high-risk: *χ^2^*= 1.832, *P* = 0.176; intermediate *vs.* high-risk:* χ^2^*= 0.134, *P* = 0.714, Fig 2E). According to our novel recurrence-risk classification, 54 cases (32.5%) and 112 cases (67.5%) in the external validation group were classified into low and high-risk groups, respectively. The RFS rates at 5 years were 87/112 (77.7%) for the high-risk group and 4/54 (7.4%) for the low-risk group. There were significant differences in the 5-year RFS rates between the low-risk group and high-risk groups (*χ^2^*= 64.956, *P* < 0.001, Fig 2F).

## Discussion

Although EORTC appears to be a useful decision-making clinical tool, one of the problems in the EORTC risk table is that the prevalence of patients at different risk classifications is disproportionate. In this study, 77.6% of patients (287 patients in the testing group; 189 patients in the validation group; 123 patients in the external validation group) were classified into the intermediate-risk group according to EORTC. Xu et al. 17, Ieda et al. 10 and Sakano et al. 18 displayed similar results with 78.0%, 87.8% and 92.5% of NMIBC cases classified as intermediate-risk, respectively. The low frequency of low-risk cases could possibly because of the lower ratio of G1 tumors in our current study (18.6% in the testing group; 20.0% in the validation group; 22.9% in the external validation group) compared with the EORTC trials (43.2%) 8. Since the other Asian studies including Japanese 10, 18-19 and Korean 20 populations also found a low incidence of G1, there might be racial difference in grade distribution of NMIBC between Asian and Caucasian populations.

Although some earlier Caucasus studies claimed significant differences in RFS rates between different risk groups 21-22, other studies (Asian or American), including ours (Fig. 2A, 2C and 2E), found that prediction of recurrence was poorly related to the EORTC 10-12. Also in another Chinese study 17, no significant difference in the RFS rates was found. In our study, we could find no significant difference between groups according to the EORTC risk group stratification both in testing and validation groups. We believe that the Han Chinese population differed significantly from the population with other ethnic background analyzed by the EORTC. These may explain why EORTC does not suitable for Asians, and these phenomena underline the urgent need for promoting current predictive models among Asians 11.

In the EORTC risk group, only 6.5% of patients received BCG treatment. Although the EORTC was widely validated and recommended by international guidelines, it claimed that disease recurrence in NMIBC patients was poorly discriminated. It is worth noting that the standard postoperative adjuvant therapy in NMIBC is the bladder instillation of chemotherapy or BCG. However, the EORTC is of little use for deciding this.

Urine tumor marker detection is a common auxiliary means for early diagnosis, recurrence monitoring and prognosis evaluation of bladder cancer because it is non-invasive and easy to use. However, there is no new consensus indicator on the risk factors for the recurrence of NMIBC, especially the lack of relevant tumor indicators in urine. Urine BLCA-4, BTA, NMP22, and CEA can be used as important markers for the diagnosis and recurrence monitoring of bladder cancer 23-30. At present, the specificity and sensitivity of BLCA-4 detection for bladder cancer diagnosis have reached a high level. More and more scholars have begun to pay attention to the clinical significance of BLCA-4 in judging the prognosis of bladder cancer 23. Zhao et al. 24 retrospective analysis of 325 patients with bladder cancer confirmed that the 5-year recurrence-free survival rate of patients with low expression of tumor tissue BLCA-4 was 89.8%. BTA, also known as complement factor H-related proteins, can interfere with the complement pathway, allowing tumor cells to evade the immune system and produce tumors 25. Bladder tumor is in contact with the basement membrane of the bladder. The tumor cells bind to the surface protein receptor of the basement membrane by secreting the base protein, thereby releasing the enzyme to destroy the basement membrane, and the resulting basement membrane fragments are aggregated into the bladder to produce BTA 26. With the increase of the staging and grading of bladder tumors, the detection level of BTA increased, the detection rate of multiple tumors was significantly higher than that of single tumors, and the initial tumors were significantly higher than the recurrent tumors 27. NMP22 is one of the members of the nuclear matrix protein, which is specifically present in urinary tract transition cells. The content of NMP22 in malignant transitional cells is about 80 times that of normal cells 28. NMP22 is mainly involved in DNA replication and transcription, RNA synthesis and gene expression regulation, and its content can be determined by detecting the amount of protein released by apoptotic cells 28. Related studies have shown that NMP22 content is positively correlated with tumor size, stage, and grade 29. CEA is a tumor-associated antigen. There is no CEA in normal urinary tract, but the urinary CEA in bladder cancer patients is significantly increased 30. In this study, we also included the above four urine markers to establish a recurrence risk model.

In this study, we developed a novel risk classification to predict recurrence for Chinese patients with NMIBC to compensate for the shortcomings of EORTC. We found significant differences in RFS rates between the groups (Fig 2B, 2D and 2F). In addition, we conducted further validation study to confirm the effectiveness of this novel recurrence-risk group stratification in the Han Chinese patients. Moreover, compared with the EORTC, our new risk classification system included the bladder instillation of chemotherapy for the first time. Currently, NMIBC's adjuvant therapy is almost intravesical infusion chemotherapy, and many guidelines recommend these therapies. Of course, this model is not a tool to determine the indications for adjuvant bladder infusion therapy. However, using our new model, we can assess the risk of recurrence, with or without these adjuvant bladder perfusion treatments. In addition, other risk factors such as obesity, smoking, occupational exposure to aromatic amine chemicals, family history of bladder cancer were also been fully considered in our new model.

In conclusion, BLCA-4, BTA, NMP22, CEA, body mass index, smoking, family history of bladder cancer, occupational exposure to aromatic amine chemicals, number of tumors, instillation of chemotherapeutic agents were found to be independent predictors for recurrence after TURBT in the Han Chinese patients with NMIBC. Our novel and simple recurrence classification may predict the recurrence risk. Further researches with more patients in a multicenter cohort are needed to validate our risk classification and to enhance the effectiveness of existing treatment for the Han Chinese patients with NMIBC.

## Supplementary Material

Supplementary figure and table.

## Figures and Tables

**Table 1:** Clinicopathological characteristics, urine biomarkers and life-history traits in the testing group, validation group and the external validation group

Variables		Testing group	Validation group	External validation group	*χ^2^*/*F*/*Z*	*P*
Number of patients		360	246	166		
Age, median (IQR)		70 (62-79)	71 (63-80)	73 (64-82)	1.943	0.442
Sex (%)					1.505	0.471
	Male	288 (80.0)	194 (78.9)	125 (75.3)		
	Female	72 (20.0)	52 (21.1)	41 (24.7)		
Body mass index (%)					0.200	0.905
	<24 kg/m^2^	211 (58.6)	148 (60.2)	100 (60.2)		
	≥24 kg/m^2^	149 (41.4)	98 (39.8)	66 (39.8)		
Hypertension (%)		65 (18.1)	41 (16.7)	33 (19.9)	0.694	0.707
Diabetes (%)		47 (13.1)	24 (9.8)	17 (10.2)	1.856	0.395
Smoking (%)		275 (76.4)	180 (73.2)	124 (74.7)	0.817	0.665
Drinking (%)		264 (73.3)	163 (66.3)	116 (69.9)	3.525	0.172
Histologic type (%)					2.022	0.396
	Bladder urothelial carcinoma	335 (93.1)	233 (94.7)	156 (94.0)		
	Bladder squamous cell carcinoma	18 (5.0)	7 (2.8)	6 (3.6)		
	Bladder adenocarcinoma	7 (1.9)	6 (2.5)	4 (2.4)		
Differentiation status (%)					0.846	0.655
	Well differentiation	326 (90.6)	220 (89.4)	146 (88.0)		
	Moderate-poor differentiation	34 (9.4)	26 (10.6)	20 (12.0)		
Depth of invasion (%)					2.206	0.373
	Ta	222 (61.7)	142 (57.7)	101 (60.9)		
	T1	106 (29.4)	74 (30.1)	50 (30.1)		
	Tis	32 (8.9)	30 (12.2)	15 (9.0)		
Metastatic status (%)					2.050	0.359
	Yes	6 (1.7)	5 (2.0)	6 (3.6)		
	No	354 (98.3)	241 (98.0)	160 (96.4)		
Grade (%)					1.516	0.863
	G1	67 (18.6)	49 (20.0)	38 (22.9)		
	G2	198 (55.0)	130 (52.8)	84 (50.6)		
	G3	95 (26.4)	67 (27.2)	44 (26.5)		
Tumour size (%)					3.053	0.217
	<3 cm	247 (68.6)	152 (61.8)	108 (65.1)		
	≥3 cm	113 (31.4)	94 (38.2)	58 (34.9)		
Number of tumours (%)					3.272	0.451
	1	194 (53.9)	145 (58.9)	94 (56.6)		
	2-7	159 (44.2)	96 (39.0)	66 (39.8)		
	≥8	7 (1.9)	5 (2.1)	6 (3.6)		
Concomitant carcinoma *in situ* (%)		27 (7.5)	21 (8.5)	10 (6.0)	0.901	0.637
2nd TURBT (%)		44 (12.2)	28 (11.4)	23 (13.9)	0.566	0.753
Creatinine corrected total BLCA-4 concentrations (mean ± SD, ng/mg)		119.3±60.9	120.6±63.5	118.6±57.0	0.254	0.776
Creatinine corrected total BTA concentrations (mean ± SD, U/mg)		9.3±5.3	9.7±5.1	9.4±4.0	0.926	0.367
Creatinine corrected total NMP22 concentrations (mean ± SD, μg/mg)		9.4±5.8	10.2±6.9	9.8±6.6	1.543	0.214
Creatinine corrected total CEA concentrations (mean ± SD, ng/mg)		2.1±1.5	1.9±1.3	2.2±0.9	1.700	0.183
Family history of bladder cancer (%)		16 (4.4)	13 (5.3)	11 (6.6)	1.109	0.574
Occupational exposure to aromatic amine chemicals (%)		157 (43.6)	117 (47.6)	80 (48.2)	1.384	0.501
BCG induction therapy (%)		69 (19.2)	60 (24.4)	41 (24.7)	3.205	0.201
Bladder instillation of chemotherapeutic agents (%)		47 (13.1)	22 (8.9)	23 (13.9)	3.112	0.211
EORTC recurrence risk classification (%)					3.308	0.391
	Low-risk	31 (8.6)	19 (7.7)	16 (9.6)		
	Intermediate-risk	287 (79.7)	189 (76.8)	123 (74.1)		
	High-risk	42 (11.7)	38 (15.5)	27 (16.3)		
Follow up period, median (IQR)		44.5 (8.5-60.0)	39.0 (7.0-60.0)	41.0 (8.0-60.0)	0.184	0.830

Abbreviation: BCG: Bacillus Calmette-Guerin; EORTC: European Organization for Research and Treatment of Cancer; IQR: interquartile range; BLCA-4: bladder cancer-specific nuclear matrix protein 4; BTA: bladder tumour antigen; NMP22: nuclear matrix protein 22; CEA: carcinoembryonic antigen.

**Table 2:** Univariate and multivariate logistic regression analyses for the NMIBC recurrence

Parameter		Univariate analysis			Multivariate analysis ^b^		
		Risk ratio (95% CI)^a^	*P*		Risk ratio (95% CI)^a^	*P*	
Age (<70 *vs.*)	≥70	1.009 (0.982, 1.038)	0.509				
Male sex (female *vs.*)	male	0.952 (0.503, 1.802)	0.881				
Body mass index (<24 kg/m^2^ *vs.*)	≥24 kg/m^2^	1.164 (1.094, 1.237)	<0.001		1.112 (1.020, 1.188)	0.007	
Hypertension (no *vs.*)	yes	1.090 (0.960, 1.250)	0.184				
Diabetes (no *vs.*)	yes	1.051 (0.557, 1.984)	0.878				
Smoking (no *vs.*)	yes	2.360 (1.555, 3.583)	<0.001		1.709 (1.528, 2.761)	<0.001	
Drinking (no *vs.*)	yes	1.786 (0.906, 3.522)	0.094				
Family history of bladder cancer (no *vs.*)	yes	1.242 (1.083, 2.398)	<0.001		1.257 (1.046, 2.862)	<0.001	
Occupational exposure to aromatic amine chemicals (no *vs.*)	yes	1.935 (1.370, 2.864)	<0.001		1.725 (1.109, 2.917)	<0.001	
Depth of invasion (Ta *vs.*)	T1, Tis	1.544 (0.763, 3.125)	0.227				
Grade (G1 *vs.*)	G2-3	1.009 (1.001, 1.019)	0.021		1.005 (0.996, 1.015)	0.257	
Histologic type (urothelial carcinoma* vs.*)	squamous cell carcinoma and adenocarcinoma	0.996 (0.988, 1.004)	0.962				
Differentiation status (well* vs.*)	moderate-poor	1.232 (0.997, 1.479)	0.084				
Metastatic status (no *vs.*)	yes	1.652 (0.861, 4.903)	0.732				
Tumour size (<3cm *vs.*)	≥3 cm	1.002 (0.996, 1.007)	0.590				
Number of tumours (1* vs.*)	≥2	1.398 (1.175, 1.821)	<0.001		1.091 (1.021, 1.165)	0.010	
Concomitant carcinoma *in situ* (no *vs.*)	yes	1.002 (0.992, 1.011)	0.762				
2nd TUR-Bt (no *vs.*)	yes	0.999 (0.997, 1.071)	0.518				
BCG induction therapy (no *vs.*)	yes	0.975 (0.904, 0.995)	0.046		0.963 (0.896, 1.120)	0.709	
Bladder instillation of chemotherapeutic agents (no *vs.*)	yes	0.817 (0.728, 0.926)	<0.001		0.874 (0.854, 0.982)	<0.001	
BLCA-4		1.633 (1.308, 2.038)	<0.001		1.381 (1.100, 1.732)	<0.001	
BTA		2.316 (1.562, 3.104)	<0.001		2.024 (1.602, 2.437)	<0.001	
NMP22		1.804 (1.392, 2.337)	<0.001		1.907 (1.425, 2.552)	<0.001	
CEA		1.265 (1.060, 1.508)	0.009		1.093 (1.034, 1.156)	0.002	

Abbreviation: BCG: Bacillus Calmette-Guerin; BLCA-4: bladder cancer-specific nuclear matrix protein 4; BTA: bladder tumour antigen; NMP22: nuclear matrix protein 22; CEA: carcinoembryonic antigen. ^a^ Note that the risk ratio corresponds to a unit increase in the explanatory variable;^ b^ The risk ratio was adjusted for all significant recurrence predictors of the univariate logistic regression analysis.

**Table 3:** The calculated performance indices for different models and our model for the testing group (n = 360).

Group	AUC	Standard error	95% CI	*P*	Sensitivity (%)	Specificity (%)
Body mass index	0.680	0.029	0.623-0.736	<0.001	73.0	61.4
Smoking	0.636	0.031	0.576-0.697	<0.001	92.6	34.6
Family history of bladder cancer	0.531	0.031	0.469-0.592	0.332	7.4	98.7
Occupational exposure to aromatic amine chemicals	0.578	0.031	0.518-0.639	0.002	52.9	62.7
Number of tumours	0.731	0.028	0.677-0.785	<0.001	65.1	69.9
Bladder instillation of chemotherapeutic agents	0.553	0.031	0.493-0.614	0.013	18.5	92.2
BLCA-4	0.804	0.023	0.759-0.849	<0.001	87.3	54.9
BTA	0.807	0.023	0.761-0.852	<0.001	80.4	69.9
NMP22	0.705	0.028	0.651-0.760	<0.001	64.0	70.6
CEA	0.780	0.025	0.731-0.828	<0.001	65.6	79.7
Model	0.907	0.016	0.876-0.939	<0.001	79.9	92.6

Abbreviation: AUC: area under the receiver operating characteristic curves; CI: confidence interval; BLCA-4: bladder cancer-specific nuclear matrix protein 4; BTA: bladder tumour antigen; NMP22: nuclear matrix protein 22; CEA: carcinoembryonic antigen.

## Abbreviations

BCG: Bacillus Calmette-Guerin
BLCA-4: bladder cancer-specific nuclear matrix protein 4
BTA: bladder tumour antigen
CEA: carcinoembryonic antigen
CIS: carcinoma *in situ*
CUETO: Spanish Urological Club for Oncological Treatment
EORTC: European Organization for Research and Treatment of Cancer
IQR: interquartile range
NMIBC: non-muscle invasive bladder cancer
NMP22: nuclear matrix protein 22
RFS: recurrence-free survival
ROC: receiver operating characteristic
TURBT: transurethral resection of bladder tumor
UICC: Union for International Cancer Control
