# Supplementary material for: Development and Validation of a Novel Recurrence Risk Stratification for Initial Non-Muscle Invasive Bladder Cancer in the Han Chinese Population
Source: J Cancer. 2020 Jan 14;11(7):1668–78. doi: 10.7150/jca.38649 (PMC7052852; doi:10.7150/jca.38649)
Supplement: Supplementary file 1 — Supplementary figure and table. [file jcav11p1668s1.pdf]

Table S1. EORTC risk group stratification of recurrence

Risk group stratification	Risk factor
Low-risk tumours	Primary, solitary, Ta, LG/G1, <3 cm, no CIS
Intermediate-risk tumours	All tumours not defined in the two adjacent categories (between the category of low and high risk)
High-risk tumours	Any of the following:<br>1. T1 tumour<br>2. HG/G3 tumour<br>3. CIS<br>4. Multiple and large (>3 cm) Ta G1G2 tumours (all conditions must be present in this point)

CIS: carcinoma *in situ*; HG: high grade; LG: low grade.

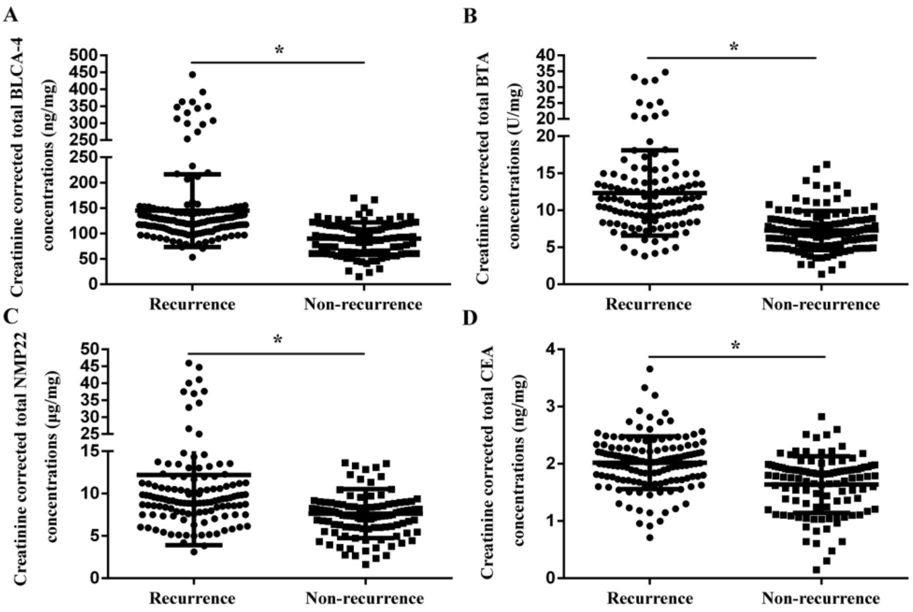

Fig. S1 Creatinine-corrected total urinary biomarker concentrations in the recurrence group and the non-recurrence group (validation group). (A) BLCA-4. (B) BTA. (C) NMP22. (D) CEA. * *P*<0.05.
